# Horizontal Gene Transfer and The Evolution of Bacterial Cooperation

**DOI:** 10.1111/j.1558-5646.2010.01121.x

**Published:** 2011-01

**Authors:** Sorcha E Mc Ginty, Daniel J Rankin, Sam P Brown

**Affiliations:** 1 Department of Biochemistry, University of ZurichBuilding Y27, Winterthurerstrasse 190, CH-8057 Zurich, Switzerland; 2 Swiss Institute of BioinformaticsQuartier Sorge Bâtiment Génopode, CH-1015 Lausanne, Switzerland; 3 University of Oxford, Department of ZoologySouth Parks Road, Oxford OX1 3PS, United Kingdom

**Keywords:** Cooperative traits, mobile genetic element, parasite, plasmid, social dilemma

## Abstract

Bacteria frequently exhibit cooperative behaviors but cooperative strains are vulnerable to invasion by cheater strains that reap the benefits of cooperation but do not perform the cooperative behavior themselves. Bacterial genomes often contain mobile genetic elements such as plasmids. When a gene for cooperative behavior exists on a plasmid, cheaters can be forced to cooperate by infection with this plasmid, rescuing cooperation in a population in which mutation or migration has allowed cheaters to arise. Here we introduce a second plasmid that does not code for cooperation and show that the social dilemma repeats itself at the plasmid level in both within-patch and metapopulation scenarios, and under various scenarios of plasmid incompatibility. Our results suggest that although plasmid carriage of cooperative genes can provide a transient defense against defection in structured environments, plasmid and chromosomal defection remain the only stable strategies in an unstructured environment. We discuss our results in the light of recent bioinformatic evidence that cooperative genes are overrepresented on mobile elements.

Microorganisms are now known to display all of the hallmarks of a complex and coordinated social life (Crespi 2001; West et al. 2006, 2007).

The production of public goods, which are costly to the producer while benefiting other members of the population, is possibly the most common form of social behavior in microbes (West et al. 2007). As such, the production of public goods is prone to the invasion of nonproducers, who gain the benefit of the public good without paying the cost of production (West et al. 2007). Such public goods can be seen in a wide range of bacterial products such as siderophore production (Griffin et al. 2004; Buckling et al. 2007) or biofilm formation (Brockhurst et al. 2006; Xavier and Foster 2007).

Bacterial genomes often contain mobile genetic elements such as conjugative plasmids or lysogenic phages (Frost et al. 2005; Thomas and Nielsen 2005). A bacterial plasmid is a species of nonessential extrachromosomal DNA that replicates autonomously as a moderately stable component of the cell's genome (Novick 1987). Plasmids can be inherited vertically, during cell division, or can be transmitted horizontally between different bacterial lineages. Plasmids may be seen as parasitic DNA and changes to the bacterial and plasmid chromosome to facilitate a reduction in the deleterious effect of the plasmid may be expected and observed over time (Bouma and Lenski 1988; Modi and Adams 1991), although it is also well-known that plasmids also carry benefits to their host (Simonsen 1991; Rankin et al., in press).

A recent study, which looked into the set of proteins expressed in 22 *Escherichia* and *Shigella* genomes, found that secreted proteins were overrepresented on mobile elements (Nogueira et al. 2009). This result seems to confirm a previous theoretical study (Smith 2001), which suggested that horizontal transfer is an important mechanism for the maintenance of cooperation in microbes. Smith's study first captured the familiar social dynamics of chromosomally determined cooperators and defectors, illustrating that a population of individuals that produced a public good could easily be invaded by individuals that did not produce it, resulting in the breakdown of the public good (Smith 2001), an outcome known as “the tragedy of the commons” (Hardin 1968; Rankin et al. 2007). Smith (2001) then demonstrated that allowing plasmids to carry the gene producing the public good could lead to the “tragedy” being averted, and cooperation maintained in the face of cheats—as the cheats would become infected with the plasmid and therefore with the cooperative gene. However, Smith's model did not allow for the fact that plasmids that did not code for the public good could also be present in the population or arise through mutation, and that they could outcompete the cooperative plasmid.

Here, we aim to examine and expand upon the model of Smith (2001) by broadening the potential strategy set of plasmids to include “null” plasmids that do not induce a cooperative phenotype in their host cells. We then further extend these models to consider a spatially structured (metapopulation) setting, as population structuring is well understood to favor cooperation (Lion and van Baalen 2008; Kummerli et al. 2009). We find that cooperative plasmids cannot withstand invasion by defector plasmids within a patch. However, they are maintained by spatial structure and cooperative plasmid carriers dominate other strains in a metapopulation (both in the absence and presence of defector plasmids) because they engineer the conditions for their own survival by virtue of their higher productivity and horizontal transmission.

## Models and Results

### WITHIN-PATCH POPULATION DYNAMICS

We start by imagining a population of defectors, the density of which is given by *D*, and introduce a rare mobile element, *m*, which contains the gene coding for cooperative behavior. We denote cells that are infected by the element as *D_m_*, and these cells are phenotypically equivalent to chromosomal cooperators (*C*). All phenotypic cooperators pay a cost *c* and confer a benefit *b* to the local population, which can be used by all *N* members of the local population. Table 1 shows the parameters used in the models. The plasmid is transmitted at a rate β, imposes a cost *v* on the host and segregates (i.e., is lost during cell division) at a rate *s*. From these rates, we can write the following equations:

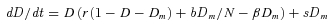
(1a)
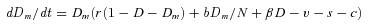
(1b)

**Table 1:** Parameters used in within-host dynamics. Estimates of parameter values in simulations are based upon previous empirical research: growth rate and carrying capacity (Brown et al. 2006b), plasmid transmission rates (Dionisio et al. 2002), and plasmid segregation (Simonsen 1991)

Parameter	Description
*N*	Total population density
*r*	Intrinsic growth rate of the individual populations
*k*	Baseline carrying capacity of the individual populations in the absence of social interaction
*b*	Benefit of cooperation
*c*	Cost of cooperation
b	Transmission rate of *m* plasmid
*v*	Cost of plasmid carriage
*s*	Segregation rate of plasmids
a	Transmission rate of *i* plasmid

In the above model (and also subsequent ones) the underlying demography of birth and death is described by the logistic growth model (*r*(1 −*N*/*k*)). All strains have the same intrinsic growth rate, *r*, and baseline carrying capacity in the absence of social interaction, *k* (here normalized to 1). *N* is the total population size (i.e., *N*=*D_m_+ D*). The strains *D* and *D_m_* are competitively equivalent (apart from their infection status and its effects) thus an increase in the number of either *D* or *D_m_* will have a negative impact on others due to competition for limiting resources (such as in the Lotka-Volterra competition model (Otto and Day 2007)). Our model assumes that the population is well-mixed and all *D* individuals have an equal chance of being infected with the *m* plasmid (at rate β) via mass action transmission (Levin et al. 1979).

Analysis of model 1 reveals that cooperative plasmid carriers can invade a pure defection equilibrium D*= 1, D*_m_= 0, if β > *s*+*v*+*c* (i.e., *R*^m^_0_ > 1). Thus cooperation can be restored by a mobile genetic element if the rate of horizontal transfer is greater than the combined costs of cooperation, carriage, and loss by segregation.

The model of Smith (2001) found that cooperative plasmid carriers (*D_m_*) could persist due to horizontal defection, thus restoring cooperation. However, bacterial populations harbor multiple types of plasmids. A second plasmid, which does not code for cooperation, could invade a population of cooperative plasmids. Here, we therefore extend the model of Smith (2001) to include the dynamics of bacterial populations infected with defector plasmids (*D_i_*), as well as cooperator (*D_m_*) plasmids. We assume that both *m* and *i* plasmids belong to the same incompatibility group, meaning that *i* plasmid carriers cannot be infected with *m* plasmids and vice versa. Susceptible (*D*) individuals are infected by the *m* plasmid at rate *h* and by the *i* plasmid at rate α.
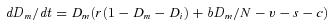
(2a)
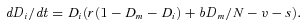
(2b)

**Figure 1:**
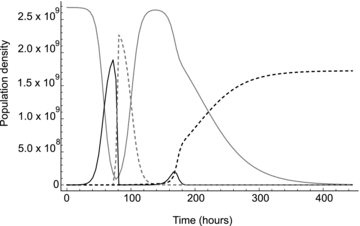
Mobile cooperation cannot be maintained in the presence of a null plasmid in an unstructured environment. Phenotypic cooperators (*C* and *D_m_*) are lost from the population but *D* is maintained at low levels through segregation of *D_m_* (initially) and subsequently *D_i_* (see model 2). We see dynamics of nontransitive competition between *C*, *D*, and *D_m_*, with *C* increasing after *D_m_* replaces *D*. Parameters used are *r*= 1.1, *k*= 2 × 10^9^, *b*= 0.55, *c*= 0.175, β= 2.75 × 10^−9^, α= 2.75 × 10^−9^, *v*= 0.15, *s*= 0.0001, *C*(*t*= 0) =*k*(*b + r – c*), *D*(*t*= 0) = 10^5^, plasmids are introduced to the population at low levels: *D_m_*(*t*= 0) = 0, *D_m(_*(*t*= 60) = 1 and *D_i_* (*t*= 0) = 0, *D_i_*(*t*= 70) = 1. *C*= gray solid line, *D*= black solid line, *D_m_*= gray dashed line, *D_i_*= black dashed line.

As we assume incompatibility between *i* and *m* plasmids, transmission rates disappear as there are no uninfecteds (*D*) in this simple model. This model has two equilibria: D*_m_= 1 +*b*−*s*−*v*−*c*, D*_m_= 0 and D*_m_= 0, D*_m_= 1 −*s*−*v*. The first equilibrium, consisting only of the cooperator plasmid, *m*, is always unstable, due to the costs of cooperation (*c* > 0). The second equilibrium (pure defector plasmid, *i*) is stable if the costs of plasmid carriage are not excessive (i.e., if *s + v* < 1). Thus interaction between the plasmids collapses to the familiar result of a chromosomal cooperator versus defector model, with the social dilemma played out among plasmids as opposed to chromosomes. As expected, cells not engaging in cooperative behavior exclude cooperative cells (Fig. 1).

**Figure 2:**
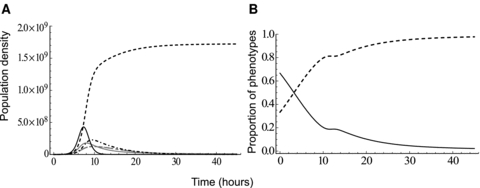
Defector phenotypes always dominate in an unstructured environment. Model and parameters as for Figure 1, except initial conditions are now an equal abundance of all strains (starting value of *C_i_*= 10^6^, whereas all other strains are set to zero). (A) *C*= gray solid line, *C_i_*= gray dashed line, *C_m_*= gray dot-dashed line, *D*= black solid line, *D_i_*= black dashed line, *D_m_*= black dot-dashed line. C, Cm, *Ci*, and *Dm* decline, *Di* dominates, *D* is maintained by segregation. (B) Proportion of the population that are phenotypic cooperators (*C, D_m_, C_m_, C_i_*) = solid line, proportion of the population that are phenotypic defectors (*D, D_i_*) = dashed line. Phenotypic defectors (*D, D_i_*) dominate.

Extending the previous analyses we can determine whether pure *D_i_* (in the limit of no segregation) is stable in this system (*D*, *D*, *C_m_*, *D_m_*, *C_i_*, *D_i_*). This pure *D_i_* equilibrium is stable provided *v* < *c*, *v* < 1 and 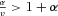, that is, *D_i_* dominates provided the cost of plasmid carriage is not prohibitive and the rate of transmission is sufficiently high. Coupled with pairwise analyses (*C*→*D*→*D_m_*→*D_i,_*, see Appendix A and models 1 and 2), this suggests the end-point of this system will be pure *D_i_* in the limit of no segregation and coexistence of *D* and *D_i_* when segregation is not negligible (Fig. 2A). Thus, although plasmid-coded cooperation can be maintained against chromosomal defectors, it is lost in the face of invasion by a noncooperative mobile element leaving a susceptible-infected scenario of susceptibles, *D*, and infecteds, *D_i_*. The complete system of all within-patch dynamics for *C, D, D_m_, D_i_, C_m_*, and *C_i_* (chromosomal cooperators and defectors infected with cooperator plasmids and defector plasmids respectively) is described in Appendix A.

#### Secondary infections: Plasmid incompatibility and multiple plasmid infections

**Figure 3:**
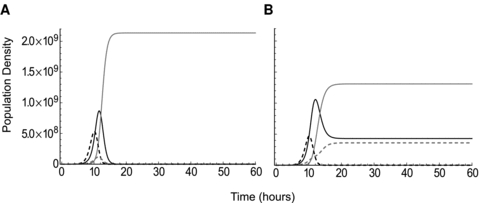
*D_im_* can invade a population of *D*, *D_i_*, and *D_m_* Compatible plasmids can infect the same cell leading to dominance of double plasmid carriers (*D_im_*) in the population, however *D_im_* decreases with increasing compatibility between the two plasmid types. *r*= 1.1, *k*= 2 × 10^9^, *b*= 0.55, *c*= .175, β= 2.75 × 10^−9^, α= 2.75 × 10^−9^, *v*= 0.15, *s*= 0.0001. *D*(*t*= 0) = 10^4^, *D_i_*(*t*= 0) = 10^4^, *D_m_*(*t*= 0) = 10^4^, *D_im_*(*t*= 0) = 10^4^ based on model above. *D_im_*= gray solid line, *D_m_*= gray dashed line, *D_i_*= black solid line, *D*= black dashed line. Panel (A) *j*= s, *Dim* invades population (B) *j*= 0.8, *D_im_* decreases with increased *j*.

In the previous section, it was assumed that there was immunity among *i* and *m* plasmid types, that is, that they belong to the same incompatibility group and therefore could not stably coexist in the same cell (Novick 1987). Two plasmids may also be prevented from existing within the same cell via a different mechanism known as surface exclusion, not modeled here (Taylor and Grant 1977; Garcillan-Barcia and de la Cruz 2008). However if *i* and *m* were to belong to different incompatibility groups then *D_i_* could be infected with *m* and *D_m_* with *i* to give *D_im_*, assuming the host cell can support two species of plasmid. *j* is a measure of the instability of the *D_im_* class, which describes the rate of decomposition of *D_im_* into *D_i_* or *D_m_*. Incompatibility between *i* and *m* increases with increasing *j*. Here, we show that *D_im_* cells can invade a population consisting of *D_i_* cells depending on the degree of incompatibility ( *j*) (Fig. 3). An analogous treatment of incompatibility via the surface exclusion mechanism yields qualitatively identical results (not shown).
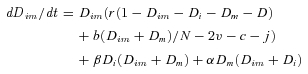
(3a)
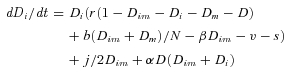
(3b)
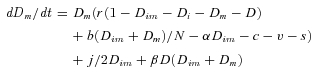
(3c)
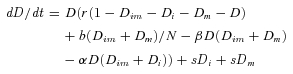
(3d)

This suggests *D_im_* can persist in the population, that is, cooperation can be restored, depending on the degree of incompatibility ( *j*) between the *i* and *m* plasmids.

When a second distinct (i.e., compatible) defector plasmid is introduced, we find that *D_ii_* cells can invade and dominate *D_im_* (see Appendix A). Therefore cooperation is not stable in this system (defection dominating among both plasmid incompatibility types). Our results therefore show the limitations in the results of Smith (2001) and demonstrate that, in the presence of null plasmids, horizontal gene transfer alone cannot maintain cooperation in an unstructured environment.

### METAPOPULATION DYNAMICS

The above model deals with mobile elements in a well-mixed, unstructured population. Recent work has shown that cooperative traits may benefit from being preferentially carried on mobile elements (Nogueira et al. 2009) so to examine cooperative plasmid dynamics in further detail we use the transitions described above to model the invasion of plasmids in a spatially structured population. This is a biologically relevant approach, particularly for bacteria, as populations tend to exhibit spatial structure, “patches” may represent different hosts, or alternatively different tissues/environments within the host. Due to the rapid growth rates of bacteria (Brown et al. 2006a; Wang and Levin 2009) we assume that the within-patch dynamics described above, take place over very short time-scales (Gilchrist and Coombs 2006), and therefore reach equilibria very quickly. Thus we introduce spatial structure via a simple Levins (1969) style metapopulation approach. Transitions in patch status are assumed to be rapid and the segregation of plasmids is ignored so that stable coexistence of types within patches can be neglected. Based on the near-dominance observed in the pairwise within-patch comparisons (Fig. 1), it is reasonable to assume that patches are either *E* (empty), or dominated by one of the cell types *C, D, D_m_, C_m_, D_i_*, or *D_m_*. Cooperator patch phenotypes are labeled *C, C_i_, C_m_*, and *D_m_*. Defector patch phenotypes are *D* and *D_i_*. It is assumed that, when resident and propagule patches are different in both cooperator and infection status, infection status is most transmissible. Thus the arrival of both *C_m_* and *D_m_* propagules in a *C* patch is assumed to generate *C_m_* patches only (at rate β, see below). Additionally it is assumed that cooperator groups are more productive (see Fig. 1) such that the colonization rate of empty patches is greater for cooperators than defectors and the extinction rate for occupied patches is greater for defector patches than cooperator patches. Table 2 describes the parameters used in the following models.

**Table 2:** Parameters used in metapopulation dynamics

Parameter	Transition	Description
*A*		Colonization rate (CR) of phenotypic cooperators
*a*_1_	*E*→*C*	CR for chromosomal cooperators
*a*_2_	*E*→*C_m_*, *E*→*D_m_*	CR of *m* plasmid carriers
*a*_3_	*E*→*C_i_*	CR of *i* plasmid carrying chromosomal cooperators
*e*		Extinction rate (ER) of occupied cooperator patches
*e*_1_	*C*→*E*	ER of chromosomal cooperators
*e*_2_	*C_m_*→*E*, *D_m_*→*E*	ER of *m* plasmid carriers
*e*_3_	*C_i_*→*E*	ER of *i* plasmid carrying chromosomal cooperators
*d*		CR of phenotypic defectors
*d*_1_	*E*→*D*	CR for chromosomal defectors
*d*_2_	*E*→*D_i_*	CR for *i* plasmid carrying defectors
*g*		ER of occupied defector patches
*g*_1_	*D*→*E*	ER of chromosomal defectors
*g*_2_	*D_i_*→*E*	ER of *i* plasmid
*x*		Rate of colonization of cooperator patches by defectors
*x*_1_	*C*→*D*	Replacement of nonplasmid carrying chromosomal cooperators by chromosomal defectors.
*x*_2_	*C_m_*→*D_i_*, *C_i_*→*D_i_*, *D_m_*→*D_i_*	Replacement of plasmid carrying phenotypic cooperators by plasmid carrying phenotypic defectors (*D_i_*).
β	*C*→*C_m_*, *D*→*D_m_*	Rate of infection of a noninfected patch by a cooperative mobile element
α	*C*→*C_i_*, *D*→*D_i_*	Rate of infection of a noninfected patch by a noncooperative (null) mobile element

#### Cooperative plasmid infection in a metapopulation (in the absence of the i plasmid)

Here, we explore whether infectious cooperative *m* plasmids can be maintained in a metapopulation. Cooperators and defectors colonize empty patches at rates *a* and *d*, respectively. Extinction of occupied cooperator patches occurs at a rate *e* and extinction of occupied cooperator patches occurs at a rate *g*. Cooperator patches are colonized by defectors at rate *x* (reflecting the cost of cooperation). As stated, cooperator patches are more productive (*a > d* and *e* < *g*, therefore overall *a/e > d/g*). Noninfected patches are colonized by mobile elements at rate β. Productivity is assumed to be determined by phenotype, cooperator or defector, as opposed to plasmid carriage.
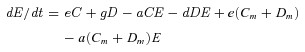
(4a)
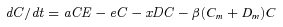
(4b)
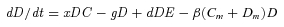
(4c)
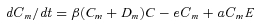
(4d)
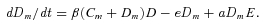
(4e)

**Figure 4:**
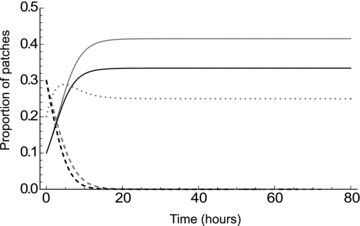
In the absence of null plasmids, mobile cooperators dominate chromosomal cooperators in a metapopulation. When the cooperative *m* plasmid can spread through the metapopulation the system tends to stable coexistence between *C_m_* and *D_m_* (in the absence of null plasmids), derived from model 4. Parameters used are *a*= 0.4, *e*= 0.1, β= 0.5, *d*= 0.3, *g*= 0.2, *x*= 0.275, *C*(*t*= 0) = 0.3, *D*(*t*= 0) = 0.3, *C_m_*(*t*= 0) = 0.1, *D_m_*(*t*= 0) = 0.1, *E*(*t*= 0) = 0.2, *C*= gray dashed line, *D*= black dashed line, *C_m_*= gray solid line, *D_m_*= black solid line, empty patches (*E*) = gray dotted line, total = black dotted line. The end-point is stable coexistence of plasmid carriers.

Cooperation cannot be maintained in this system by pure chromosomal cooperation when β > 0 (as *C*→*C_m_*) but it can be maintained by the manipulative plasmid *m* (Fig. 4). A state of pure *m* in a neutral mix of *C_m_* and *D_m_* (E*=*e*/a, C*= 0, D*= 0, C*_m_= 1 −*e*/*a*−*D_m_*) is always stable if 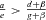. This will be the case under the assumption that *a*/*e* > *d*/*g*. Furthermore, if it assumed that there is some redundancy cost to *C_m_* (as it is both a chromosomal and a plasmid cooperator) the conclusion will be a metapopulation consisting of *E* (empty patches) and *D_m_* (plasmid cooperators). Thus all occupied patches will be cooperative due to carriage of the sole mobile element *m*, and the Smith (2001) model holds across a metapopulation (Fig. 3).

#### Social dilemmas among plasmids

There is likely to be competition at the plasmid level between the *i* and *m* plasmids, specifically in an *E, D_i,_ D_m_* model, a scenario resulting from low rates of segregation and the redundancy of chromosomal cooperation when plasmids can infect both cooperators and defectors. We model this as
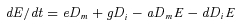
(5a)
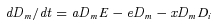
(5b)
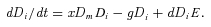
(5c)

Here, in a metapopulation, the result is qualitatively different from that in an unstructured environment (where cooperation is driven to extinction in the presence of defectors). Given *a* > *e*, *D_m_* can be maintained in the environment, either on its own (when 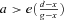) or in a state of coexistence with defector and empty patches. The coexistence equilibrium of the system is
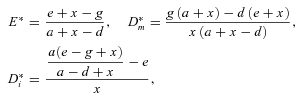
which is stable when 
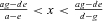
. Alternatively defectors may exist on their own when 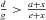.

Within a patch, plasmid-coded cooperator and defector dynamics (model 1) recapture those of chromosomal cooperators and defectors. Likewise, we see that the metapopulation dynamics for empty patches, *E*, and chromosomal cooperator, *C*, and defector, *D*, patches (Appendix B) are mirrored above by model 5. Thus, under our assumptions, it appears that the long-term metapopulation behavior is not affected by whether cooperation is encoded chromosomally (and competing with chromosomal defectors) or by a plasmid (and competing with null [noncooperative] plasmids). Under both coding systems, the cooperative variant is maintained via kin selection (Hamilton 1964; Maynard-Smith 1964; Nogueira et al. 2009).

To more fully understand the dynamics of cooperative plasmids in spatially structured populations we have modeled the complete system (*C*, *D*, *C_m_*, *D_m_*, *C_i_*, *D_i_*) for this scenario (model 6 below), the parameters for which listed in Table 2. Although this model is too complex to resolve analytically we can obtain insight into the dynamics using simulations.
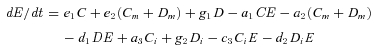
(6a)
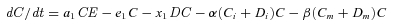
(6b)
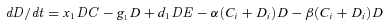
(6c)
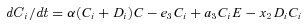
(6d)
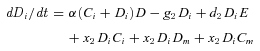
(6e)
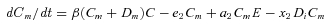
(6f)
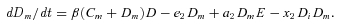
(6g)

**Figure 5:**
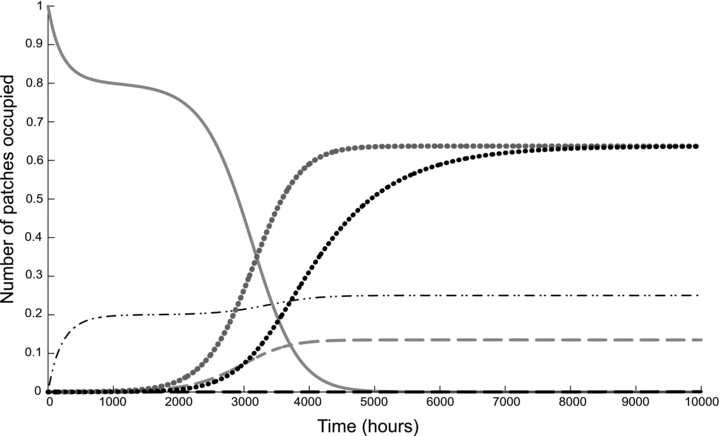
Mobile cooperation can dominate a metapopulation in the presence of null plasmids under certain conditions. Derived from model 6. We assume the cost of plasmid carriage is borne in a reduced colonization rate (*a*_2_, *a*_3_ < *a*_1_ and *d*_2_ < *d*_1_) and the cost of cooperation is less than the rate of plasmid transmission. Parameters used are *x*_1_=*x*_2_= 0.15, α=β= 0.3, *e*_1_=*e*_2_= 0.1, *g*_1_=*g*_2_= 0.2, *d*_1_= 0.35, *d*_2_= 0.3, *a*_1_= 0.5, *a*_2_=*a*_3_= 0.4. *E*= black dot-dashed line, *C*= gray solid line, *D*= black solid line, *C_i_*= gray dashed line, *D_i_*= black dashed line, *C_m_*= gray dotted line, and *D_m_*= black dotted line.

Here, we assume that the rate of spread of the plasmid within a patch that is, the rate at which plasmid carriers displace established nonplasmid carriers, is greater than the rate at which phenotypic cooperators are displaced by phenotypic defectors (i.e., the change from *D* to *D_i_* patches is greater than from *C* to *D* patches, due to infection being faster than displacement). This condition, combined with the higher productivity of phenotypic cooperators (*a* > *d*), allows mobile cooperation to dominate a metapopulation provided the *i* plasmid does not have a higher rate of transfer (Fig. 5). This cannot occur if the cooperative plasmid has a lower rate of transfer than the noncooperative plasmid (not shown).

**Figure 6:**
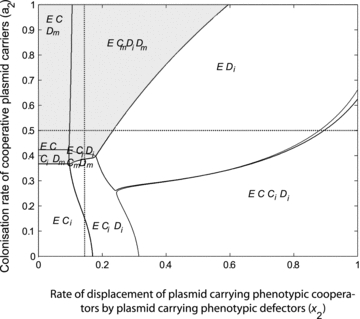
Mobile cooperation can persist in a structured environment. Derived from model 6. Parameters used are *x*_1_= 0.15, α=β= 0.3, *e*_1_=*e*_2_= 0.1, *g*_1_=*g*_2_= 0.2, *d*_1_= 0.35, *d*_2_= 0.3, *a*_1_= 0.5, *a*_3_= 0.4. *a*_1_ and *x*_1_ are marked with dotted lines. Patch values exceeding a minimum threshold value of 10^−3^ are shown. Gray shading indicates areas where mobile cooperation genes can persist.

In Figure 6, we see that the cooperative plasmid *m* can persist even when it is more vulnerable to local replacement by its cheat (plasmid *i*) than are chromosomal cooperators (by *D*) (*x*_2_ > *x*_1_, Fig. 6). This is most readily the case when cooperative plasmids have a countervailing advantage of chromosomal cooperators in colonization (*a*_2_ > *a*_1_, Fig. 6), but we also see a narrow window of *m* plasmid persistence when the plasmid cooperators are at a double disadvantage to chromosomal cooperators (*x*_2_ > *x*_1_ and *a*_2_ < *a*_1_, Fig. 6). Although this does not demonstrate that mobile cooperation is definitely at an advantage, it does show that different conditions can favor different forms of cooperation in a metapopulation (i.e., that plasmid and chromosomal cooperation are not equivalent) and that, in certain cases, mobile cooperation may be a more successful strategy (Figs. 5 and 6).

Thus, although any new chromosomally coded cooperative trait will be outcompeted by noncooperating strains within a patch, certain settings (such as the absence of null plasmids) can allow the spread of a cooperative trait on a plasmid within a patch (model 1) and also within a metapopulation (models 4 and 5) in agreement with Smith (2001). However, the cooperator plasmid remains vulnerable to usurpation by a null plasmid (particularly within a patch in which null plasmids outcompete it) but it has an advantage over null plasmids in a metapopulation in terms of establishment within empty patches as cooperators are more productive than defectors (colonization of cooperator patches is greater than that of defector patches, i.e., *a > d*).

## Discussion

Our results show that plasmid-borne cooperation is not evolutionarily stable in well-mixed populations (Fig. 1). Here, we have rephrased the model of Smith (2001) and rederive the same results, demonstrating that cooperation can be restored by infection of defectors with a manipulative mobile genetic element carrying the social trait (i.e., cooperation). The dynamics observed are similar to those of an infectious disease; the mobile element can only spread in the population when its basic reproductive number, *R_0_*, is greater than one. Smith (2001) states that his model shows that horizontal transfer of genes for such social traits is a novel mechanism for the evolutionary maintenance of cooperation. However, the results presented here suggest that this does not represent the full story.

In the short term, we do indeed see cooperation restored by the mobile element in the absence of competitor null plasmids, but longer term dynamics suggest that this is not a stable end-point. Our analyses of well-mixed populations suggest that, once introduced, a “null” or noncooperative mobile genetic element can outcompete the cooperative mobile element (Fig. 1) and all other strains (Fig. 2). The interaction between mobile cooperators and mobile defectors is a repeat of the familiar interaction between chromosomal cooperators and chromosomal defectors, that is, the social dilemma repeats itself at a lower level. The end-point is also the same: defectors outcompete cooperators both chromosomally and on plasmids. When incompatibility is removed and a second level infection is allowed the above pattern repeats itself, first *D_im_* becomes dominant but it is then replaced by *D_ii_* (models 3 and A2). These models suggest that, contrary to Smith's statement, horizontal transfer of cooperative traits cannot function as a stable mechanism for the evolutionary maintenance of cooperation within patches as these plasmids are vulnerable to being outcompeted by plasmids not coding for cooperation. The end result is that cooperation is not stable within a well-mixed patch, regardless of whether a cooperative gene is found on a chromosome or a plasmid, and whether cooperative and noncooperative plasmids are incompatible.

It has been suggested that, in the long term, beneficial genes cannot be maintained on plasmids as they will ultimately be sequestered onto the host chromosome and the plasmid will then be unable to persist (Bergstrom et al. 2000). If the cooperative trait here is considered a beneficial trait, by the above logic it might be expected that the mobile cooperative gene would be replaced by a chromosomal coding variant and thus the expectation of greater prevalence of cooperative genes on mobile elements would fail. However, that is not likely here as cooperation does not benefit the individual in a within-patch scenario as *C* is always defeated by *D* and cannot invade *D_i_* thus removing any advantage to the incorporation of the cooperative gene onto the chromosome.

In contrast to Smith's (2001) results, cooperation appears to be doomed within an unstructured environment, regardless of whether it is carried on the chromosome or on a plasmid. However, spatial structure (as in a metapopulation of patches) can promote the persistence of chromosomal cooperation (Nowak and May 1992; Griffin et al. 2004; Hauert and Doebeli 2004; Harrison and Buckling 2007; Lion and van Baalen 2008; Kummerli et al. 2009), as cooperators are then more likely to profit from interacting with other cooperators (i.e., structuring increases relatedness) if competition is sufficiently global. This is confirmed in our models, where we show that cooperation can persist in a metapopulation, both when it is coded chromosomally and on a plasmid. The metapopulation dynamics presented here are similar to models used to model the evolution of sexual and asexual lineages, where asexual lineages win out in local dynamics, but sexual lineages win out in between-patch competition (Maynard-Smith 1976; Nunney 1989; Kokko et al. 2008). We find that although the metapopulation dynamics between cooperators and defectors are the same for chromosomal or plasmid borne cooperation (*E*, *C*, *D* and *E*, *D_m_*, *D_i_*) when competing with chromosomal variants in a metapopulation mobile cooperation can become the dominant strategy (model 6), which supports the view of Nogeuira et al that there may be an advantage to cooperative traits being mobile.

A limiting assumption of our metapopulation study is that of within-patch homogeneity. Nogueira et al. (2009) assume heterogeneous patches and examine the effect upon patch relatedness of gene mobility. The “relatedness” of two individuals can be defined as the increased probability (compared to population average) of them sharing a common allele at the focal locus, in this case a locus governing a social interaction (Frank 1998). They found that horizontal transfer of a cooperative trait increases relatedness at the mobile loci, therefore favoring maintenance of cooperation through kin selection (Hamilton 1964). In other words, mobile elements act to homogenize patches (at the mobile loci), and therefore to conform more closely to the “patch homogeneity” assumptions of our metapopulation model. By allowing for variable degrees of homogeneity (varying relatedness), Nogueira et al.'s (2009) results suggest that cooperative alleles may indeed be more successful when carried on mobile elements, a conclusion supported by their sequence data. Microbial social traits often involve the secretion of an external protein, such as a public goods (West et al. 2007) and Nogueira et al. (2009) predicted and, through statistical analysis, found support for the hypothesis that secreted proteins (and thus cooperative traits) should be more often encoded on mobile elements. However, their models neglect ecological dynamics: for example the costs and benefits of cooperation are not addressed. An idea for future work in this area would be to incorporate within-patch diversity (seen in Nogueira et al. 2009) into more ecologically explicit models of plasmid dynamics and evolution (such as in the current study). This would give a more complete and realistic view of the population dynamics of mobile genetic elements and their role in bacterial social dilemmas.

Our models have focused on bacterial traits that function as public goods. The production of extracellular iron-scavenging molecules (siderophores) is an excellent example of a cooperative trait necessary for virulence in bacterial infections (West and Buckling 2003; Harrison et al. 2006), and low relatedness has been shown to lead to decreased virulence in such infections (Harrison et al 2006). In fact many other traits associated with growth and virulence in pathogenic bacteria seem to be cooperative and subject to kin selection including biofilms and immune suppression (Brown et al. 2002; Griffin et al. 2004). Understanding the link between bacterial cooperation and virulence can in principle contribute to new medical intervention strategies For example, the ability of plasmids to spread rapidly within patches opens the possibility for their use as therapeutic tools. They could be introduced to a population of pathogenic bacteria to undermine the stability of the focal population and/or drive medically useful alleles (e.g., antibiotic sensitivity) into the focal population resulting in increased success of secondary mechanisms of control, for example, antibiotics (a “Trojan Horse” strategy, Brown et al. 2009). However, as with all medical strategies for dealing with rapidly evolving pathogens such as bacteria, it is important to consider that bacterial hosts may develop resistance to plasmid uptake or that such plasmids may mutate and lose their capacity to be useful. The dynamics of such resistance are not modeled here but certainly this is an aspect which should not be neglected in future work if plasmids are to be envisaged as some form of therapy.

### CONCLUSIONS

In summary, this study has shed further light on the role of mobile genetic elements in bacterial social evolution, illustrating that mobile cooperator advantage is at best only transitory in an unstructured environment. In a structured environment, it appears that mobile cooperation can be dominated by virtue of its transmission and the high productivity of cooperators. Although previous models (Smith (2001)) have suggested that horizontal transfer of genes for cooperative traits can maintain cooperation within patches, the results presented here suggest that a cooperative plasmid will always be vulnerable on a local scale to usurpation by a null plasmid as the social dilemma repeats itself at the plasmid level.

## Appendix A: Detailed Within-population Dynamics

### THE COMPLETE SYSTEM: *C, D, C_m_, D_m_, C_i_*, AND *D_i_*

The complete system is complex and involves infection of both chromosomal cooperator and defector strains with both plasmids *i* and *m*. Plasmids are assumed to be incompatible. This gives a system of six different strains.

(A1a)

(A1b)

(A1c)

(A1d)

(A1e)

(A1f)

From this, in the absence of all other types, a pairwise comparison of chromosomal cooperators (*C*) and defectors (*D*) shows that pure chromosomal cooperation (with equilibrium population density of *C**= 1 +*b*−*c*, while defectors are absent, *D**= 0) is always unstable with respect to chromosomal defectors, whereas pure defection (when *C**= 0 and *D**= 1) is always stable, although less productive than the pure cooperation equilibrium.

For the case of plasmids, given the establishment of a pure *D**_*i*_ equilibrium (at *D**_*i*_= 1 −*s*−*v*), we now ask what happens if we allow for plasmid segregation (where plasmids are lost from infected cells at some positive rate, *s*). When *D* emerges from the infected class (positive segregation) the model becomes a representation of a classic host–parasite system with SI dynamics and vertical transmission (Anderson and May 1979). *D* individuals are generated through segregation and are susceptible to infection with *i*. Recovery is through segregation of the plasmid to give *D* once more. The pure uninfected defectors equilibrium (*D**= 1, *D**_*i*_= 0) is stable if α*> s + v*, that is when the transmission rate of plasmid *i* is high enough to compensate for the cost of plasmid carriage and loss through segregation that is, the parasitic mobile element can persist.

If segregation is negligible the Jacobian matrix for this system can be evaluated at the pure *D_i_* equilibrium from model 4 (in the limit of no segregation) such that *C**= 0, *D**= 0, *C**_*m*_= 0, *D**_*m*_= 0, *C**_*i*_= 0, and *D**_*i*_= 1 −*v*. This pure *D_i_* equilibrium is stable provided *v* < *c*, *v* < 1 and . Increasing the cost borne by *C_m_* to 2*c* (for carrying the cooperative trait twice) does not qualitatively affect these results.

### INCOMPATIBILITY AND SECONDARY INFECTIONS

Can another distinct (i.e., compatible) plasmid *a*, with a defector phenotype *i*, invade to give *D_ia_* and dominate *D_im_*? In terms of phenotype *D_ia_* can be written as *D_ii_*. Again, neglecting *s* (and *j*), we have

(A2a)

(A2b)

Pure *D_im_* (*D**_*ii*_= 0, *D**_*im*_= 1 +*b*− 2*v*−*c*) is always unstable whereas pure *D_ii_* (*D**_*ii*_= 1 − 2*v*, *D**_*im*_= 0) is stable when *v* < 1/2 (i.e., if viable). Therefore cooperation is not stable in this system.

### MODELING THE PROCESS OF SEGREGATION: DEPENDENCE ON GROWTH OR POPULATION DENSITY?

If we consider the dynamics of plasmids as akin to that of an infectious disease we consider a population of susceptibles, *S*, and infected, *I*, we have the following dynamics;

(A3a)

(A3b)

When *I* is rare and *S* is at equilibrium (i.e., *S*= K*), the condition for invasion of *I* is β*> v + c* and therefore is independent of segregation provided all density dependent growth is affected by segregation.

Alternatively we may write

(A3c)

(A3d)

if segregation affects only the maximum growth rate (i.e., *r*) and is independent of density dependence.

Expanding these equations, we can now see that

(A3e)

(A3f)

Rescaling *s*′, we call our scaled segregation rate *s*=*rs*′, we write

which gives

(A3g)

(A3h)

the method by which we model segregation in our model. As segregation occurs during reproduction, it will affect the density-independent part of bacterial growth. Thus, both segregation *s* and growth *r* are in the same dimensions, meaning they can be factored together. We therefore assume that segregation is scaled so that, as long as segregation is less than growth (i.e., *r* > *s*), it can be assumed that segregation is linked to growth. This way of modeling segregation is consistent with previous models that examine similar issues (Stewart and Levin 1977; Bergstrom et al. 2000; Lili et al. 2007).

## Appendix B: Further Metapopulation Dynamics

### CHROMOSOMAL COOPERATION IN A METAPOPULATION

Here we focus on cooperator (*C*), defector (*D*) and empty patches (*E*) only.

(B1a)

(B1b)

(B1c)

This system will either tend to an equilibrium of pure empty patches (*E**= 1, *C**= 0, *D**= 0) or one of three occupied states depending on the parameter values:

1. Defectors are excluded from the system (pure cooperation); (*E**=*e*/*a*, *C**= 1 −*e*/*a*, *D**= 0). This is stable when .
2. Cooperators are excluded from the system (pure defection); (*E**=*g*/*d*, *C**= 0, *D**= 1 −*g*/*d*). This is stable when .
3. Coexistence between cooperators and defectors within the structured environment (, ) which is stable when .

### COOPERATIVE PLASMID INFECTION IN A METAPOPULATION

This system has numerous equilibria, the relevant ones in terms of dynamics are:

1. Pure *C*: *E**=*e*/*a*, *C**= 1 −*e*/*a*, *D**= 0, *C**_*m*_= 0, *D**_*m*_= 0. This is never stable when β > 0.
2. Pure *D*: *E**=*g*/*d*,*C**= 0, *D**= 1 −*g*/*d*, *C**_*m*_= 0, *D**_*m*_= 0. This is never stable as *d*/*g* < *a*/*e*.
3. Pure *m* (a neutral mix of *C_m_* and *D_m_*): see main text.

### SOCIAL DILEMMAS AMONG PLASMIDS

The results mirror those for chromosomal cooperation within a metapopulation (see model B1 above).
